# Morphological and molecular identification of the cat flea *Ctenocephalides felis* from Bangladesh

**DOI:** 10.5455/javar.2025.l894

**Published:** 2025-03-26

**Authors:** Md Shamsudduha, Md Mahfuzur Rahman, Jannatun Naher, Azizul Islam Barkat, Sumaiya Akter, Mohammad Shamimul Alam

**Affiliations:** Department of Zoology (Genetics and Molecular Biology Branch), University of Dhaka, Dhaka, Bangladesh

**Keywords:** Cat flea, *Ctenocephalides felis*, ectoparasite, molecular identification, morphological identification, *16S rRNA*

## Abstract

**Objective:**

The present study was designed to conduct molecular and morphological identification of cat fleas (*Ctenocephalides felis*) from Bangladesh along with nucleotide polymorphism and phylogenetic analysis.

**Materials and Methods:**

Samples were collected from two hosts (cat and human). The species was identified through morphological studies first, and then DNA was extracted for subsequent molecular analysis. A part of the mitochondrial *16S rRNA* gene was amplified by polymerase chain reaction using extracted DNA as a template. The amplified region was sequenced using the Sanger dideoxy method. The sequence was subjected to NCBI BLASTn search. BioEdit and MEGA 11 software were used for multiple sequence alignment (MSA) and generating a phylogenetic tree.

**Results:**

Morphological features such as shape, size, and appendages showed similarity with *C. felis.* The metatibial formula of chaetotaxy (2-2-2-2-1-3) was confirmed for species-level identification. NCBI BLASTn search showed the highest sequence identity with the available sequence of *C*.* felis* such as 99.78% (NC_049858.1) and 99.12% (MW420044.1 and MK941844.1) with 100% query coverage. MSA of *C*.* felis *sequences from different geographical distributions show their sequence affinities with each other, and the phylogenetic tree presents their relationship with each other.

**Conclusion:**

Both morphological and molecular studies clearly indicate the identity and confirmation of cat flea (*C*. *felis)* from Bangladesh.

## Introduction

Cat flea *Ctenocephalides felis* (Bouché 1835) is a member of the *Pulicidae* family under the Order Siphonaptera. They are important ectoparasites with veterinary medical significance considered to be the most common species of flea on earth [1]. These are hematophagous ectoparasites that transmit a variety of zoonotic vector-borne pathogens such as *Rickettsia felis*, *Bartonella clarridgeiae, B*. *henselae*, and *Yersinia pestis* which are the etiologic agents of flea-borne spotted fever, cat scratch disease, and plague, respectively [2–6]. It also acts as a secondary host of a cestode parasite, *Dipylidium caninum,* and its bite develops Flea Allergic Dermatitis (FAD) in cats, dogs, and humans [7–9]. Though the cat flea infests mainly domesticated cats and dogs worldwide, it is also found on other domesticated and feral animals such as domesticated sheep, donkeys, goats, water buffalo, roof rats, Rüppell’s fox, and so on, and its adaptability to various habitat conditions has made it geographically widespread [1,10–12]. Cat fleas may occasionally infest humans, resulting in discomfort and skin irritation [9,12,13].

In Bangladesh, *R*.* felis* infections vectored by fleas are diagnosed throughout the country [14]. Cat fleas play a potential role in transmitting *R. felis* (a bacteria) to humans globally, particularly in Bangladesh [15]. Although several morphological and molecular studies on cat fleas are conducted worldwide [16–18], only morphology has been studied in Bangladesh [19]. Dog flea *C*.* canis* and the cat flea *C. felis* have often been misidentified based on morphology [20]. Therefore, for this tiny organism, DNA-based molecular methods could provide reliability in species identification [21,22]. Very few morphological studies [19] and the absence of significant molecular analysis for identifying cat fleas in Bangladesh urged further studies to combine molecular techniques with morphology. In the present study, we utilized the *16S rRNA* gene of mitochondrial DNA for the identification of the species from Bangladesh.

## Materials and Methods

### Ethical approval

No animal experiment has been done in this experiment. However, the fleas were collected from the animals without harming or giving minimum disturbances, following the animal welfare standards.

### Sample collection and morphology analysis

Several specimens (about 10) of cat fleas were collected from each of the two hosts, a human and a cat. All specimens of cat fleas were collected from two locations on the Dhaka University campus, Dhaka, Bangladesh (23°43’52’’N, 90°23’29’’E and 23°43’28.31’’N, 90°24’03.82’’E). They were identified as *C. felis* based on external morphology. Due to morphological similarities, two samples were chosen for further study. Each was carried in a separate Eppendorf tube to the Genetics and Molecular Biology lab for morphometric and molecular study. Both specimens were photographed with a stereomicroscope (Leica EZ4). For morphological studies, the existing literature was consulted [16,17,19]. After confirmation of the identification of the species according to the morphological studies, one of the samples was sacrificed for the molecular studies.

### DNA extraction, PCR amplification, sequencing, and bioinformatics information

The CTAB DNA extraction method was used for the isolation of DNA from the sample. A previously reported primer pair suitable for insects was used in PCR amplification for the mitochondrial *16S rRNA* gene [23]. The forward and the reverse primers were (5’-CGC CTG TTT AAC AAA AAC AT-3’) and (5’-TTT AAT CCA ACA TCG AGG-3’), respectively. The purified PCR product was sequenced by the Sanger dideoxy method. After receiving the sequencing file, it was visualized and edited using the software FinchTV. Noisy areas of the sequence, as seen in the chromatogram, were removed from both ends to base the subsequent analyses on a quality sequence. The edited sequence was used for species identification and the comparison among related sequences with the aid of the Basic Local Alignment Search Tool (BLASTn) of the National Center for Biotechnology Information (NCBI). Multiple sequence alignment (MSA) of the species from different geographical areas was performed with the aid of ClustalW in BioEdit software [24]. DNA sequences were analyzed using both BioEdit [24] and MEGA 11 software [25] to find polymorphic sites.

Molecular phylogenetic trees were constructed using MEGA 11 software [25]. Two neighbor-joining phylogenetic trees were made and analyzed, one utilizing the *16S rRNA* region and the other using the *CO1* region of mitochondrial DNA. Various species belonging to the Siphonaptera order were selected for phylogenetic analysis. Both phylogenetic trees were constructed with the same species to facilitate a comparative analysis of their phylogenetic relationships. The partial mitochondrial *16S rRNA* sequence from our research specimen was incorporated into the construction of the *16S rRNA* tree. Fruit fly (*Drosophila melanogaster)* was used as an outer group for both the tree. The bootstrap value was 100 for this study.

## Results and Discussion

### Morphological identification

The flea was initially identified as *C*.* felis *based on its morphology before conducting molecular analysis.

### Generic morphological features

**Figure 1.**
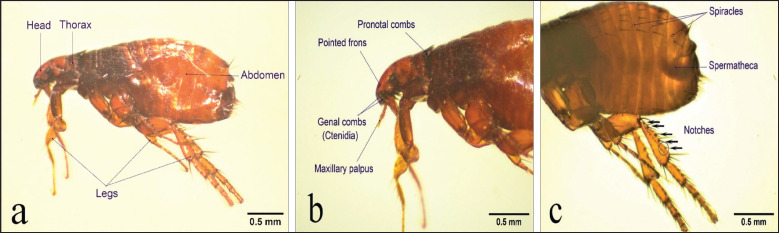
Female *C*.* felis*. (a) The left side of the whole body. (b) Head and thorax region, showing pointed frons; 1^st^ genal ctenidial length exceeds half of the 2^nd^ one; pronotal combs; and maxillary palpus. (c) Leg’s tibia bearing 5 notches; female spermatheca (a holding organ); tergal spiracles.

The specimen is a small, 2.9 mm wingless insect with a shiny surface and a dark or reddish-brown coloration (Fig. 1a). At the posterior or ventral edges of the head, there are rows of dark spines, ctenidia, or comb that faced backward. The pronotal and genal ctenidium on the head both have eight or nine horizontally aligned spines (Fig. 1b).

The sternum consists of one or two ventral spines; the tergum of the ninth abdominal segment is changed to form the clasper, which is absent in females but present in males [19]. The third pair of legs is far longer than the other two pairs utilized for jumping (Fig. 1a). Each leg has a coxa, femur, tibia, and tarsus, as well as pygidium and antepygidial bristles on the posterior end. The female has spermatheca as a holding organ, and the forehead has maxillary palpus ventrally (Fig. 1c) [19].

### Species-specific morphological features

The species *C*.* felis* is distinguished by its notably angled frons and more pointed-looking head (Fig. 1b). Length is usually more than double the height of the head. The first spine of the genal ctenidia exceeds at least half of the second in length [16]. *Ctenocephalides felis *often has two setae in its lateral metanotal area (LMA) [16]. It has small tergal spiracles as shown in Figure 1c. It contains fewer bristles in comparison with other species in this genus [16]. All six legs’ tibiae have five to six notches. One thick bristle between the post-median and apical long bristles [19]. The metatibial formula of chaetotaxy is 2-2-2-2-1-3. Female (the present specimen) spermatheca (a holding organ) contains a short hilla (Fig. 1c) [19].

**Table 1.** BLASTn search results.

Description	Max score	Total score	Query cover (%)	E value	Identical (%)	Accession
*Ctenocephalides felis* isolate EL2017- DRISC mitochondrion, complete genome	828	828	100%	0.0	99.78%	NC_049858.1
*Ctenocephalides felis felis* mitochondrion, complete genome	813	813	100%	0.0	99.12%	MW420044.1
*Ctenocephalides felis felis* mitochondrion, complete genome	813	813	100%	0.0	99.12%	MK941844.1
*Ctenocephalides felis* 16S ribosomal RNA gene, partial sequence; mitochondrial	706	706	90%	0.0	98.03%	GQ387498.1
*Ctenocephalides canis* mitochondrion, complete genome	767	767	100%	0.0	97.34%	NC_063710.1
*Ctenocephalides orientis* isolate F5 mitochondrion, complete genome	745	745	100%	0.0	96.46%	NC_073009.1

**Figure 2.**
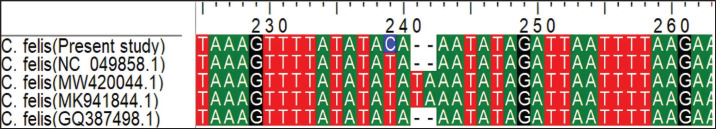
A partial view of the MSA of *16S rRNA* region of *C*.* felis *from different countries. The scale on top represents site numbers.

### Molecular identification

After the successful extraction of DNA, a partial region of the mitochondrial *16S rRNA* gene was amplified using the polymerase chain reaction (PCR) technique. The amplification was accomplished using the primers specific for the mitochondrial *16S rRNA* gene. The resulting PCR products were visualized through agarose gel electrophoresis. The size of the amplified region was 472 base pairs (bp). Based on quality, a 451 bp DNA region was selected for further analysis (Accession no. OR708655). Mitochondrial markers are used in the identification and phylogenetic studies of different species, including insects [26]. *16S rRNA* gene is one of the mitochondrial markers that is commonly used in the identification of different animal species [26,27].

**Table 2.** Polymorphic sites of cat fleas originated from different countries.

Name (Accession number)	Country	Sites				
		51	239	241	242	395
*Ctenocephalides felis* (Present study)	Bangladesh	A	C	–	–	A
*Ctenocephalides felis* (MW420044.1)	China	G	T	T	A	A
*Ctenocephalides felis* (MK941844.1)	China	G	T	T	A	A
*Ctenocephalides felis* (NC_049858.1)	USA	A	T	–	–	A
*Ctenocephalides felis* (GQ387498.1)	USA	A	T	–	–	–
Gap (–) represents base deletion.						

**Table 3.** Nucleotide base pair study of the sample specimen and other related fleas.

Name (Accession number)	Total number of				Percentage of GC (GC%)
	Adenine (A)	Thymine (T)	Guanine (G)	Cytosine (C)	
*Ctenocephalides felis * (Present study)	186	164	63	38	22.4%
*Ctenocephalides felis* (NC_049858.1)	186	165	63	37	22.2%
*Ctenocephalides felis* (MW420044.1)	186	166	64	37	22.3%
*Ctenocephalides felis* (MK941844.1)	186	166	64	37	22.3%
*Ctenocephalides canis* (NC_063710.1)	188	161	64	38	22.6%
*Ctenocephalides orientis* (NC_073009.1)	190	158	66	38	23.0%
*Pulex irritans * (NC_063709.1)	177	165	68	38	23.7%

More than 99% sequence similarity has been found in the case of *16S rRNA* gene fragment of the present specimen when aligned with *C*.* felis* of the previous study [28]. NCBI BLAST nucleotide search shows 99.78% similarity with accession number NC_049858.1 and 99.12% similarity with accession number MW420044.1 and MK941844.1 where query coverage was 100% (Table 1). Other species of the *Ctenocephalides* genus such as *C*.* canis *and *C*.* orientis *show 97.34% (NC_063710.1) and 96.46% (NC_073009.1) similarity, respectively (Table 1). The sequence of our sample has been submitted to GenBank of the National Center for Biotechnology Information (NCBI). The GenBank accession number of our sample sequence is OR708655. The result of MSA, as outputs of ClustalW and BioEdit [24], has been presented in Figure 2.

### Analysis of polymorphic sites

Aligning the sequence of the Bangladeshi sample with that of other countries provided information about polymorphic sites. Single nucleotide polymorphism (SNP), insertion, or deletion (indel) of nucleotide bases at the specific site of *16S rRNA* gene of *C*.* felis* from different countries are shown in Table 2.

*Ctenocephalides felis* of China with accession numbers MW420044.1 and MK941844.1 contain Guanine (G) at site 51 whereas all others contain Adenine(A) at the same site (Table 2). The Bangladeshi specimen of the present study is distinct from all others at site 239 where there is Cytosine (C) instead of Thymine (T) (Table 2). *C*.* felis* of China (MW420044.1, MK941844.1) has a specific “TA” insertion at sites 241–242 whereas all others possess deletion on this site. GQ387498.1 has a deletion of a base at site 395 while others contain Adenine (A) as shown in Table 2.

A comparison of the nucleotides in related species of *C*.* felis* has been presented in Table 3. The sample specimen of the present study contains 41.24% Adenine (A), 36.36% Thymine (T), 13.97% Guanine (G), and 8.43% Cytosine (C) in the selected *16S rRNA* region. The percentage of A is the highest, and C is the lowest. The AT/GC ratio of the sample specimen was found to be 3.47.

### Phylogenetic analysis

**Figure 3.**
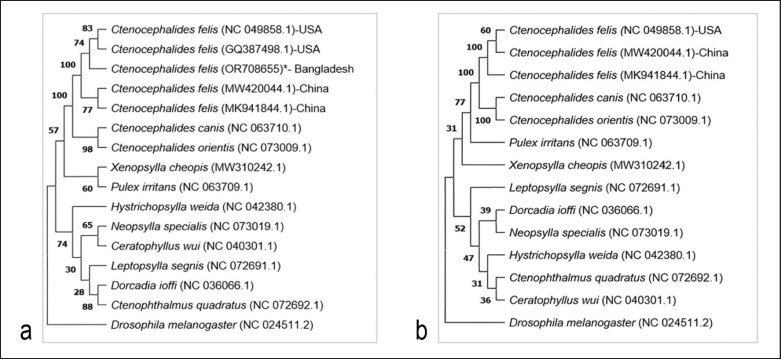
Phylogenetic relationship among different species from the order Siphonaptera using mitochondrial *16S rRNA* gene (a) and mitochondrial *CO1* gene (b). *Marked sequence depicts the present study. Numbers representing bootstrap values.

Neighbor-joining phylogenetic trees were constructed based on the sequences of the *16S rRNA* (Fig. 3a) and *CO1* genes (Fig. 3b) to understand the relationships between the target flea species and other fleas belonging to the Siphonaptera order. A total of 16 partial sequences from the mitochondrial *16S rRNA* region (Fig. 3a) and 14 partial sequences from the *CO1* region (Fig. 3b) of the same 11 species were taken for phylogenetic relation analysis. *Drosophila melanogaster* (Accession number: NC_024511.2) was used as an outgroup that falls outside the group of interest.

In the mitochondrial *16S rRNA* gene-based phylogenetic tree, *C*.* felis *of Bangladesh (OR708655) shows a closer relationship with that of the USA (NC_049858.1, GQ387498.1) when compared to the sequences from China (MK941844.1, MW420044.1) (Fig. 3a). Thus, it indicates that the USA origin seems to be more closely related to Bangladeshi origin compared to China.

In the case of both *16S rRNA *and *CO1*, the species *C*.* felis* of different origins construct a cluster in the phylogenetic tree (Fig. 3a). One of the two *C*.* felis* specimens of China (MW420044.1) shows more relatedness with the USA (NC_049858.1) as shown in the phylogenetic tree based on the mitochondrial *CO1* region (Fig. 3b). The relationship of USA, China and Bangladesh origin cannot be ascertained due to a lack of *CO1* sequence data from Bangladesh. *C*. *canis* (NC_063710.1) and *C*.* orientis* (NC_073009.1) cluster together and are separated from *C*.* felis* in both mitochondrial *16S rRNA* (Fig. 3a) and *CO1* gene (Fig. 3b) based phylogenetic tree.

*Xenopsylla cheopis* (MW310242.1) and *Pulex irritans* (NC_063709.1) are in the same clade in the *16S rRNA* tree (Fig. 3a) but separated in the *CO1* tree (Fig. 3b). The cluster of *Neopsylla specialis* (NC_073019.1) and *Ceratophyllus wui* (NC_040301.1) is connected to the cluster of *Leptopsylla segnis *(NC_072691.1), *Dorcadia ioffi* (NC_036066.1), and *Ctenophthalmus quadratus* (NC_072692.1); *Hystrichopsylla weida* (NC_042380.1) is in a separate branch (Fig. 3a). On the other hand, in the *CO1* tree, the cluster of *D. ioffi* (NC_036066.1) and *N. specialis *(NC_073019.1) is connected with cluster of *C. quadratus *(NC_072692.1)*, C. wui* (NC_040301.1), and *H. weida *(NC_042380.1); *L. segnis *(NC_072691.1) remains in a separate branch (Fig. 3b).

## Conclusion

The integration of both molecular and morphological approaches provides a comprehensive and reliable method for the accurate identification of this important ectoparasite. *C*.* felis* of different geographical locations showed distinctiveness in the sequence of the *16S rRNA* gene, which may help in strain identification as well. To the best of our knowledge, our sequence of *C. felis* from Bangladesh is the first to be submitted to NCBI GenBank. Besides, the study can aid in further studies on the biology, ecology, and control of cat fleas as well as contribute to our understanding of their potential role in disease transmission.

## List of abbreviations

BLAST, Basic local alignment search tool; CO1, Cytochrome Oxidase 1; DNA, Deoxyribonucleic acid; MEGA, Molecular Evolutionary Genetics Analysis; NCBI, National Center for Biotechnology Information; PCR, Polymerase chain reaction; RNA, Ribonucleic acid.
